# Myofibroblastome extramammaire de localisation pelvienne: à propos d’un cas

**DOI:** 10.11604/pamj.2021.38.154.28060

**Published:** 2021-02-11

**Authors:** Kanta Ka, Mamadou Lassana Foba, Sidy Ka, Mamadou Moustapha Dieng, Papa Macoumba Gaye, Ahmadou Dem

**Affiliations:** 1 Service de Radiothérapie, Centre Hospitalier National Universitaire Dalal Jamm, Guédiawaye, Sénégal,; 2 Université Cheikh Anta Diop de Dakar, Dakar, Sénégal,; 3 Service de Chirurgie Plastique, Centre Hospitalier Universitaire Aristide Le Dantec, Dakar, Sénégal,; 4 Service d'Oncologie, Centre Hospitalier Universitaire Aristide Le Dantec, Dakar, Sénégal

**Keywords:** Myofibroblastome, extra mammaire, radiothérapie, tumeur rare, à propos d’un cas, Myofibroblastoma, extramammary, radiotherapy, rare tumor, case report

## Abstract

Un myofibroblastome de type mammaire est une tumeur molle rare; les myofibroblastomes extramammaires sont particulièrement rare. Un homme de 78 ans s'est présenté en consultation pour des douleurs pelviennes soulagées par la défécation ou les urines. Le toucher rectal retrouve une masse en avant de la paroi rectale antérieure. L'imagerie par résonance magnétique (IRM) montre une masse de 10 x 6 x 8cm, bien circonscrite et hétérogène, située en arrière de la vessie qu'elle refoule vers l'avant, en avant du recto-sigmoïde. L'immunohistochimie montre des cellules tumorales co-exprimant CD34 et la desmine de façon diffuse, expression de Rb dans la majorité des cellules, expression des récepteurs aux œstrogènes, expression intense et diffuse de la P16, un index de prolifération avec le ki67 estimé à 25%. Il n'y a pas eu de récidive après 8 mois de radiothérapie d'induction suivie de chirurgie. Un myofibroblastome de type mammaire est une tumeur rare et bénigne. La récidive n'est quasiment pas observée après traitement local. Ce cas permet de mettre en avance la possibilité d'utiliser la radiothérapie afin de faciliter la chirurgie.

## Abstract (en)

Mammary myofibroblastoma is a rare soft-tissue tumor. Extramammary myofibroblastomas are particularly rare. We here report the case of a 78-year-old man presenting with pelvic pain relieved by defecation or urination. Rectal examination showed a mass in front of the anterior rectal wall. The magnetic resonance imaging (MRI) showed a well-circumscribed and heterogeneous mass measuring 10 x 6 x 8cm located behind the bladder which was pushed forward in front of the rectosigmoid. Immunohistochemical analyses showed diffuse co-expression on CD34 cells and desmin, Rb expression on most cells, oestrogen receptor expression, intense and diffuse P16 expression and a ki67 proliferation index of 25%. The patient had no recurrence 8 months after radiotherapy followed by surgery. Breast myofibroblastoma is a rare and benign tumor. Recurrence is hardly observed after local treatment. This study highlights the supporting role of radiotherapy in the efficacy of surgery.

## Introduction

Décrits pour la première fois dans le sein par Wargotz *et al*. en 1987 [1], les myofibroblastomes mammaires sont des tumeurs bénignes rares. La localisation extra mammaire est plus fréquemment rapportée. Le site habituel est inguinal [2,3]. Nous rapportons ce cas suivi et traité dans notre service.

## Patient et observation

**Figure 1:**
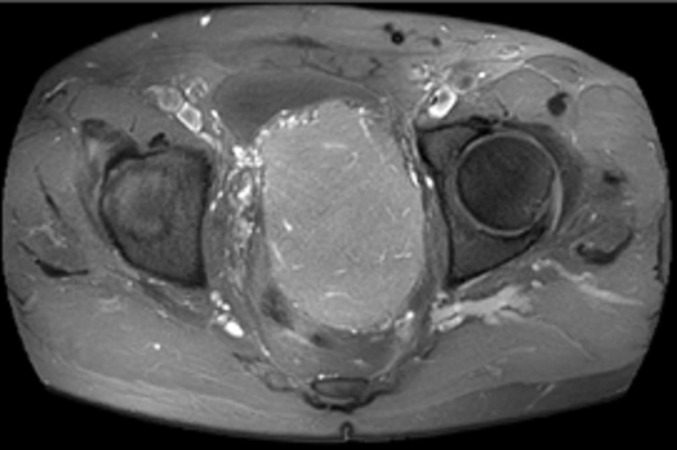
IRM axiale du pelvis en T2, masse pelvienne refoulant la vessie en avant et le rectum en arrière

**Figure 2:**
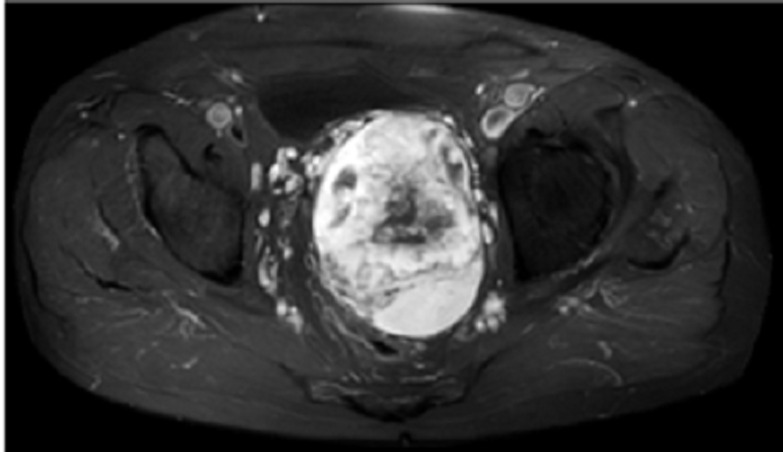
IRM axiale T1 Gadolinium, masse pelvienne

**Figure 3:**
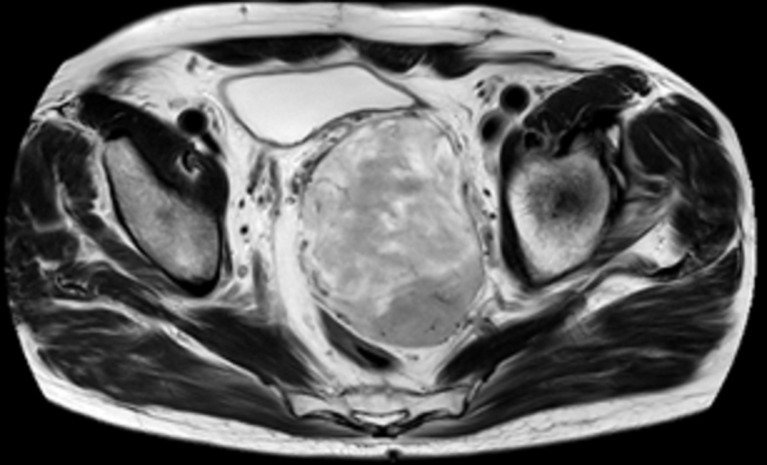
IRM axiale T2 pelvis, masse hétérogène

**Figure 4:**
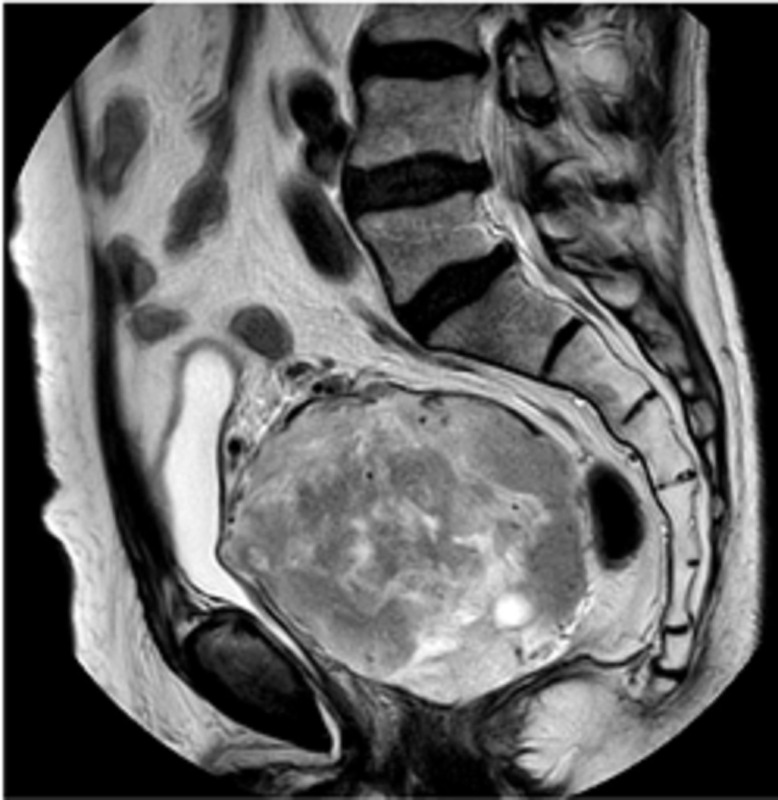
IRM sagittale T2 pelvis, masse hétérogène occupant tout le pelvis

Notre patient est âgé de 78 ans, aux antécédents de cancer de la prostate traité par chirurgie il y a 14 ans et pose d´un sphincter artificiel urinaire pour fuite urinaire, présente depuis un an une asthénie après l´exonération qui s´aggrave progressivement jusqu´à être décrite en épuisement. Plus récemment, une sensation de masse pelvienne et douleur des membres. Le toucher rectal retrouve une masse. L´IRM montre une masse de 10 x 6 x 8cm, bien circonscrite et hétérogène, située en arrière de la vessie qu´elle refoule vers l´avant, en avant du recto-sigmoïde. L´immunohistochimie montre des cellules tumorales co-exprimant CD34 et la desmine de façon diffuse, expression de Rb dans la majorité des cellules, expression des récepteurs aux œstrogènes, expression intense et diffuse de la P16, un index de prolifération avec le ki67 estimé à 25%. Il n´y a pas eu de récidive au bout de 8 mois de suivi post-radiothérapie d´induction suivie de chirurgie (Figure 1, Figure 2, Figure 3, Figure 4).

## Discussion

La description du myofibroblastome a été faite pour la première dans le sein [1]. La localisation extramammaire est rare. Ils surviennent le plus souvent de la lignée de lait embryonnaire, comme les seins accessoires [4]. McMenamin a décrit un cas de myofibroblastome abdominal [4]. La région inguinale est la localisation préférentielle et rarement, l´aisselle, la région paratesticulaire [4], la vésicule séminale [5], et la région périanale [6]. Il s´agit d´une tumeur retrouvée de façon préférentielle chez l´homme âgé, la femme en postménopause, les enfants et adolescents [1]. La moyenne d´âge des patients atteints de myofibroblastome extramammaire selon Abdul-Ghafar est de 52,5 ans [2]. La douleur est rarement décrite comme symptomatologie. Le diamètre des plages de myofibroblastome extramammaire décrit est de 1 à 4 cm [1,4]. Généralement, il s´agit d´une tumeur bien délimitée et de couleur blanche à grise. Une consistance caoutchouteuse avec une fine capsule et les zones hémorragiques ont également été décrites [7].

Le myofibroblastome est compose d´amas fasciculaires abondantes de cellules fusiformes et ovales qui sont disposées en tourbillons [3]. L´échographie montre une échogénicité mixte dans le myofibroblastome [8]. Le scanner permet de faire un diagnostic différentiel et voir l´extension aux organes de voisinage [3]. Le scanner ainsi que l´IRM sont utiles pour le diagnostic de myofibroblastome [4]. Le myofibroblastome est iso-intense en T1 et hypo intense pondéré en T2 [9]. Cette description est similaire à celle de l´IRM de notre patient. La tumeur est généralement positive pour l´actine des muscles lisses, l´actine spécifique aux muscles et vimentine, négative pour c-kit, antigène carcinoembryonnaire, S-100, mélanome humain noir-45 (HMB-45) et à la membrane latente du virus Epstein Barr protéine 1 [3,9,10]. Les myofibroblastomes extramammaire sont faiblement positifs pour la desmin et le CD34 [3,9].

Dans le cas présent l´immunohistochimie montre des cellules tumorales co-exprimant CD34 et la desmine de façon diffuse, expression de Rb dans la majorité des cellules, expression des récepteurs aux œstrogènes, expression intense et diffuse de la P16, un index de prolifération avec le ki67 estimé à 25%. Les myofibroblastomes peuvent être confondus avec un certain nombre de tumeurs dont le sarcome de Kaposi, le léiomyosarcome, le schwannome intra-nodal, le léiomyosarcome métastasé bénin (BML), le sarcome folliculaire à cellules dendritiques (FDCS), la tumeur myofibroblastique inflammatoire (IMT), le mélanome malin métastatique et le carcinome métastatique [7].

L´herpès humain virus 8 (HHV8) est souvent impliqué dans le sarcome de kaposi. Il s´agit d´une tumeur vasculaire limite atteignant la peau et les muscles et dans les stades avancés, les ganglions lymphatiques. Contrairement au sarcome de Kaposi [11], les myofibroblastomes sont de localisation mammaire le plus souvent et liés à une anomalie de la lignée lactée embryonnaire. Le léiomyosarcome est une entité des sarcomes des tissus mous [12]. C´est une tumeur mésenchymateuse maligne d´origine musculaire lisse, ce qui n´est pas le cas des myofibroblastomes. Le schwannome intra-nodal désigne des tumeurs survenant dans les ganglions lymphatiques, reclassés comme des myofibroblastomes en palissade [13,14]. La similitude réside dans le fait que ces deux tumeurs sont positives à la vimentine et S100 et la différence se fait sur la négativité pour le SMA et le CD34 dans les myofibroblastomes.

Le léiomyosarcome bénin métastasé est rare, de localisation utérine et métastase souvent au niveau des poumons [15]. L´hystérectomie est le traitement de référence. Il se différencie des myofibroblastome de par la localisation. Le sarcome folliculaire à cellules dendritiques (FDCS) a été décrit par de nombreux termes tels que lymphome, sarcome, néoplasme histiocytaire [16]. Il est positif à CD21, CD35 et/ou CD23, vimentine, fascine, HLA-DR, EMA, D2-40, clusterin et CXCL13 [17]. L´IMT est principalement situé dans les poumons, puis dans l´abdomen et d´autres parties telles que le foie, la vessie et l´estomac. C´est une tumeur à potentiel invasif, métastatique et récidivante [18]. La présentation clinique dépend de sa localisation [19]. Les myofibroblastome sont bien circonscrits contrairement au mélanome malin métastatique et carcinome.

## Conclusion

Les myofibroblastomes extra mammaire sont rares, bénins. La chirurgie est le traitement de référence. L´objectif de la radiothérapie d´induction est de réduire la masse pour faciliter la chirurgie. Le risque de récidive est très faible. Le diagnostic différentiel est difficile du fait de sa rareté.
